# Ts2631 Endolysin from the Extremophilic *Thermus scotoductus* Bacteriophage vB_Tsc2631 as an Antimicrobial Agent against Gram-Negative Multidrug-Resistant Bacteria

**DOI:** 10.3390/v11070657

**Published:** 2019-07-18

**Authors:** Magdalena Plotka, Malgorzata Kapusta, Sebastian Dorawa, Anna-Karina Kaczorowska, Tadeusz Kaczorowski

**Affiliations:** 1 Laboratory of Extremophiles Biology, Department of Microbiology, Faculty of Biology, University of Gdansk, 80-822 Gdansk, Poland; 2 Department of Plant Cytology and Embryology, Faculty of Biology, University of Gdansk, 80-308 Gdansk, Poland; 3 Collection of Plasmids and Microorganisms, Faculty of Biology, University of Gdansk, 80-308 Gdansk, Poland

**Keywords:** lytic enzyme, Peptidoglycan recognition proteins (PGRPs), peptidoglycan, *Pseudomonas aeruginosa*, *Acinetobacter baumannii*

## Abstract

Bacteria that thrive in extreme conditions and the bacteriophages that infect them are sources of valuable enzymes resistant to denaturation at high temperatures. Many of these heat-stable proteins are useful for biotechnological applications; nevertheless, none have been utilized as antibacterial agents. Here, we demonstrate the bactericidal potential of Ts2631 endolysin from the extremophilic bacteriophage vB_Tsc2631, which infects *Thermus scotoductus*, against the alarming multidrug-resistant clinical strains of *Acinetobacter baumannii*, *Pseudomonas aeruginosa* and pathogens from the Enterobacteriaceae family. A 2–3.7 log reduction in the bacterial load was observed in antibacterial tests against *A. baumannii* and *P. aeruginosa* after 1.5 h. The Ts2631 activity was further enhanced by ethylenediaminetetraacetic acid (EDTA), a metal ion chelator (4.2 log reduction in carbapenem-resistant *A. baumannii*) and, to a lesser extent, by malic acid and citric acid (2.9 and 3.3 log reductions, respectively). The EDTA/Ts2631 combination reduced all pathogens of the Enterobacteriaceae family, particularly multidrug-resistant *Citrobacter braakii*, to levels below the detection limit (>6 log); these results indicate that Ts2631 endolysin could be useful to combat Gram-negative pathogens. The investigation of *A. baumannii* cells treated with Ts2631 endolysin variants under transmission electron and fluorescence microscopy demonstrates that the intrinsic antibacterial activity of Ts2631 endolysin is dependent on the presence of its N-terminal tail.

## 1. Introduction

The emergence and spread of multidrug-resistant (MDR) bacteria is a global public health threat that imperils the effectiveness of medical treatments for many infectious diseases, even in the most developed countries [1]. Antimicrobial resistance places a tremendous burden on healthcare systems and society, with an annual cost estimated by the European Medicine Agency to be €1.6 billion in the European Union member states [2,3]. It seems that the demand for new antimicrobials has never been higher, and many efforts have been made to develop novel, alternative therapies against bacterial pathogens resistant to conventional treatments [4,5,6,7]. From this perspective, there has been a great concern about the alarming rise in antibiotic resistance in the Gram-negative bacteria *Acinetobacter baumannii* and *Pseudomonas aeruginosa*, which belong to the ESKAPE group pathogens (*Enterococcus faecium, Staphylococcus aureus, Klebsiella pneumoniae, Acinetobacter baumannii, Pseudomonas aeruginosa,* and *Enterobacter* species); these pathogens are the leading cause of nosocomial infections worldwide [8]. *A. baumannii* is a rapidly emerging pathogen, especially in intensive care units, and causes urinary tract infections, meningitis, pneumonia, bacteraemia, and wound infections [9]. In 2017, the World Health Organization listed *A. baumannii* as a critical priority for combating antibiotic-resistant bacteria [10]. The WHO indicated that the second critical pathogen is *P. aeruginosa*, an aetiological factor of bacteraemia, ventilator-associated pneumonia (VAP), urinary tract infections, and skin/soft tissue infections [11]. Both *A. baumannii* and *P. aeruginosa* are naturally resistant to many antibiotics due to the presence of an outer membrane (OM), which is basically impermeable to drugs and has a higher predominance of efflux pumps [1]. Infections due to these bacteria, especially carbapenem-resistant strains of *A. baumannii* (CRAB) and *P. aeruginosa* (CRPsA), are associated with high mortality [10,12,13,14].

In recent years, we have witnessed the development of novel therapeutic strategies to combat MDR pathogens based on phage lytic enzymes, such as endolysins [15]. Endolysins, also known as peptidoglycan (PGN) hydrolases, are enzymes produced by most dsDNA bacteriophages (viruses that infect bacteria) at the end of their life cycle to facilitate phage progeny release from the host bacteria [16]. The successful external use of endolysins, initially against Gram-positive bacteria and currently against Gram-negative pathogens, made them potent antimicrobial agents [6]. Endolysins cleave the glycosidic and/or amide bonds in a peptidoglycan, which is made of glycan strands cross-linked by short peptide stems and forms the bag-shaped structure surrounding the cytoplasmic membranes of almost all bacteria [17]. Endolysins are classified into five groups based on their specificity and act as muramidases, glucosamidases, amidases, endopeptidases, and carboxypeptidases [18]. Among them, N-acetylmuramoyl-l-alanine amidases are the earliest and most frequently identified peptidoglycan hydrolases [19].

Recently, in our laboratory, we discovered and characterized novel endolysins derived from *Thermus scotoductus* bacteriophages: Ph2119 [20] and vB_Tsc2631 [21,22]. These are two of the few known endolysins from thermophilic bacteriophages and are rare examples of thermostable amidases. The 2013 database of the endolysins encoded in dsDNA phage genomes consisted of 629 proteins [23] and included only six endolysins derived from thermophilic bacteriophages; only one of these was predicted to act as an amidase. While the search for endolysins with novel domain modules continues [24], especially in the genomes of uncultured bacteriophages, none of the 2628 putative endolysins identified from nearly 200,000 uncultured viruses showed any similarity to the bacteriophage endolysins that infect bacteria from the genus *Thermus*.

Thermophiles are organisms that thrive in extreme conditions, such as high temperature, pressure, and extreme pH or salt concentrations [25]; hence, their enzymes are often capable of withstanding high temperatures and generally exhibit increased resistance to denaturation and proteolysis [26]. These features made them immensely useful in applications requiring or involving processing at high temperatures, such as PCR [27,28,29,30,31].

The enormous stability and endurance of proteins of a thermophilic origin may be exploited for other applications, especially that mesophilic endolysins considered as perspective antibacterial agents may face stability problems. This can be exemplified by LysK endolysin that kills *Staphylococcus aureus*. Due to the changes in the endolysin secondary structure, LysK dramatically loses stability at temperatures above 40 °C, which significantly affects the efficacy of the enzyme [32,33].

Thermostability of our newly identified Ts2631 endolysin and the bactericidal activity of Ts2631 towards *Thermus* bacteria prompted us to pursue the Ts2631 potential against alarming MDR Gram-negative bacteria, such as MDR clinical strains of *A. baumannii*, *P. aeruginosa*, and MDR enterobacteria. The availability, known safety, and proven action of many compounds, such as EDTA, malic acid, and citric acid, which weaken the OM of Gram-negative bacteria, encouraged us to apply them as additives in antibacterial tests of Ts2631 endolysin.

## 2. Materials and Methods 

### 2.1. Bacterial Strains and Growth Conditions

Chemically competent *Escherichia coli* DH5α and BL21(DE3) cells (Thermo Fisher Scientific, Waltham, MA USA) were prepared to maximize the transformation efficiency and recombinant protein expression, respectively. Bacteria were cultivated at 37 °C in Luria Broth (LB) medium with shaking. MDR clinical strains of *A. baumannii*, *E. coli*, *Citrobacter freundii*, *Citrobacter braakii*, *K. pneumoniae*, and *Enterobacter cloacae* were kindly provided by Dr. Marek Bronk from the Department of Clinical Microbiology at University Clinical Centre, Gdansk, Poland with patterns of antibiotic resistance provided for each strain. All strains were deposited in the Collection of Plasmids and Microorganisms (KPD) at the University of Gdansk under the following accession numbers: *Acinetobacter baumannii* KPD 205, *Acinetobacter baumannii* KPD 581, *Escherichia coli* KPD 217, *Citrobacter freundii* KPD 219, *Citrobacter braakii* KPD 218, *Klebsiella pneumoniae* KPD 298, and *E. cloacae* KPD 297. *Pseudomonas aeruginosa* KPD 430, *Pseudomonas aeruginosa* KPD 431 with their antibiotic resistance patterns were provided by the KPD. *Thermus scotoductus* MAT2631 was kindly provided by MATIS, Iceland and *Thermus thermophilus* HB8 DSM 579 was purchased from Leibniz Institute DSMZ-German Collection of Microorganisms and Cell Cultures. Bacteria belonging to the genus *Thermus* were grown on *Thermus* 162 medium (no 878 on the list of recommended media for microorganisms of Leibniz Institute DSMZ) shaking at 60 °C.

### 2.2. Protein Purification

The plasmid pLT1 (a derivative of the pET15b vector, Ap^R^) was used for the overproduction of Ts2631 endolysin [21]. Mutant Ts2631Δ2–22 was prepared previously in our laboratory [22] with the QuikChange II Site-Directed Mutagenesis Kit (Agilent Technologies, Santa Clara, CA, USA), according to the manual. The Ts2631Δ2–22 deletion variant was prepared by removing the N-terminal region of the protein RILEPWNRWYRQKRAYRVRLT (positively charged residues are underlined). Plasmid pRARE (Cm^R^) served as a source of tRNAs for rare *E. coli* codons (Merck KGaA, Darmstadt, Germany). *E. coli* BL21(DE3)[pRARE] cells carrying the expression plasmids were cultivated at 37 °C in 500 mL LB to an OD_600_ of 0.4–0.5. The overproduction of Ts2631 endolysin and its Δ2–22 variant was induced with 1 mM isopropyl-β-d-thiogalactopyranoside (IPTG) for 4 h at 37 °C. Overproduced proteins were purified from bacterial lysates on TALON cobalt metal affinity resin according to the manufacturer’s procedure for bath/gravity-flow column purification (Takara Bio Europe AB, Goteborg, Sweden). Proteins bound to the TALON resin were eluted with 150 mM imidazole in NPi buffer (50 mM NaH_2_PO_4_, pH 8.0, 300 mM NaCl, 0.1% Triton X-100, 10% [*vol/vol*] glycerol), and pooled fractions containing pure proteins (as judged by SDS-PAGE) were dialysed against buffer D (25 mM potassium phosphate buffer, pH 8.0, 50 mM KCl, 0.1% Triton X-100, 50% glycerol, and 0.1 mM ZnSO_4_). A Bradford assay was used to determine the protein concentration [34]. From 1 litre of *E. coli* BL21(DE3)[pLT1] culture, we were able to obtain 18 mg of a homogeneous enzyme preparation, which was immediately frozen in liquid nitrogen and stored at −80 °C for further analysis.

### 2.3. Lytic Activity of Ts2631 Endolysin

The turbidity reaction assays were performed in a 96-well plate format by measuring the decrease in the OD_600_ values over 20 min period (with 0.5 min intervals) in an EnSpire Multimode Plate Reader (Perkin Elmer, Waltham, MA, USA). Each reaction was conducted in triplicate and consisted of 190 μL of viable cells suspended in 10 mM potassium phosphate buffer, pH 8.0 (K/PO_4_) and 10 μL of tested proteins diluted in K/PO_4_ buffer to a final concentration of 1.23 µM. Negative controls with K/PO_4_ buffer were subtracted from the sample measurements. All of the reactions, controls and samples with Ts2631 endolysin, were performed in the presence of the same volume of protein storage buffer D. The lytic activity was calculated as follows [ΔOD_600_ sample (endolysin added) − ΔOD600 (buffer only)]/initial OD_600_. The statistical significance was calculated by using two-tailed Student’s unpaired *t* test with GraphPad Prism 5.0 software (GraphPad, San Diego, CA, USA). In control reactions, the OMs of Gram-negative *T. thermophilus* HB8 and *T. scotoductus* MAT2631 bacteria were permeabilized by chloroform treatment as described previously [20]. Briefly, the bacteria were grown until the late-exponential phase and then centrifuged at 4500× *g* for 15 min at 4 °C. The OMs of the harvested cells were permeabilized by gentle shaking with chloroform-saturated 50 mM Tris-HCl, pH 7.7 for 45 min at room temperature (for 1 litre of cell culture, 100 mL of 50 mM Tris-HCl, pH 7.7 and 100 mL of chloroform were added). The permeabilized Gram-negative cells were then washed, suspended in 50 mL of 10 mM potassium phosphate buffer at pH 8.0, aliquoted, and frozen at −80 °C. Prior to use, the OD_600_ of the suspended cells was adjusted to 1.0.

### 2.4. Transmission Electron Microscopy

Ts2631 endolysin (final concentration of 7.4 µM) was mixed with 10^8^ of *T. thermophilus* HB8 or *A. baumannii* CRAB KPD 205 cells in a volume of 500 µL. Samples were incubated for 1.5 h at 60 °C and 37 °C, respectively and fixed in 2.5% glutaraldehyde in phosphate-buffered saline (PBS, Sigma-Aldrich, St. Louis, MO, USA) and then postfixed in 1% osmium tetroxide in PBS. Following ethanol dehydration, samples were embedded in Epon resin, cut on a Leica UC7 ultramicrotome, and contrasted in uranyl acetate and lead citrate. Transmission electron microscopy studies were performed using an FEI Tecnai BioTwin Spirit microscope (FEI Company, Hillsboro, OR, USA).

### 2.5. Antibacterial Assays

*In vitro* antibacterial assays were performed with purified Ts2631 endolysin. Clinical strains of *A. baumannii*, *E. coli*, *C. freundii*, *C. braakii*, *K. pneumoniae*, and *E. cloacae* were grown at 37 °C in LB medium until an OD_600_ of 0.5 was reached (When indicated *A. baumannii* cells were grown to OD_600_ = 1.2). The bacteria were centrifuged (4000× *g*, 15 min, 20 °C), washed, and suspended in potassium phosphate buffer with a pH of 8.0. Approximately 10^6^ (100-fold dilution) or 10^7^ (10-fold dilution when indicted) of cells were mixed with 50 µg of Ts2631 endolysin dissolved in potassium phosphate buffer to make a final volume of 100 µL and a final concentration of 25 μM. For experiments with the addition of OM permeabilization agents, EDTA, citric acid, and malic acid were included in the reaction mixtures at final concentrations of 0.5, 0.36, and 0.6 mM, respectively. In all cases, negative controls with the phosphate buffer mixed with Ts2631 endolysin storage buffer D (25 mM potassium phosphate buffer pH 8.0, 50 mM KCl, 0.1% Triton X-100 and 50% glycerol) in the presence or absence of EDTA, malic acid, and citric acid were performed. For assessing the activity of Ts2631 endolysin in the presence of serum, 10 mM potassium phosphate buffer, pH 8.0 was replaced by 1× Fetal Bovine Serum (Biowest, Nuaillé – France). The mixtures were incubated in Eppendorf tubes at 37 °C for 1.5 h, and appropriate dilutions were spread onto LB agar plates. Colonies were counted after an overnight incubation at 37 °C. The antibacterial activity was quantified as the relative inactivation in logarithmic units (= log_10_ (N_0_/N_i_), were N_0_ = number of untreated cells (in the negative control) and N_i_ = number of treated cells counted after incubation). Average ± standard deviations for all experiments are given for *n* = 3 repeats.

### 2.6. Fluorescence Microscopy

Antibacterial assays for fluorescence microscopy were prepared as described above, with 10^8^ *A. baumannii* cells mixed with Ts2631 endolysin or its Δ2–22 variant (final concentrations of 7.4 µM). Cells incubated with 10 mM potassium phosphate buffer, pH 8.0 and storage buffer D, served as the negative control. To visualize the membranes of bacteria, SynaptoRed C2 (N-(3-triethylammoniopropyl)-4-(6-(4-(diethylamino)phenyl)hexatrienyl)pyridinium dibromide) was used (Sigma-Aldrich, St. Louis, MO, USA). Bacterial nucleoids were stained with DAPI (4′, 6′-diamidino-2-phenylindole) as described elsewhere [35]. Stained bacteria (3 μL) were immobilized on a 1% agarose pad, placed on a glass microscope slide and examined with a Nikon Eclipse E800 (epifluorescence microscope combined with differential interference contrast, DIC). Images were collected and processed with Lucia Laboratory Imaging Software (Laboratory Imaging, s.r.o., Praha, Czech Republic).

## 3. Results

### 3.1. Activity of Ts2631 Endolysin against Viable Bacteria from the Thermus Genus

**Figure 1:**
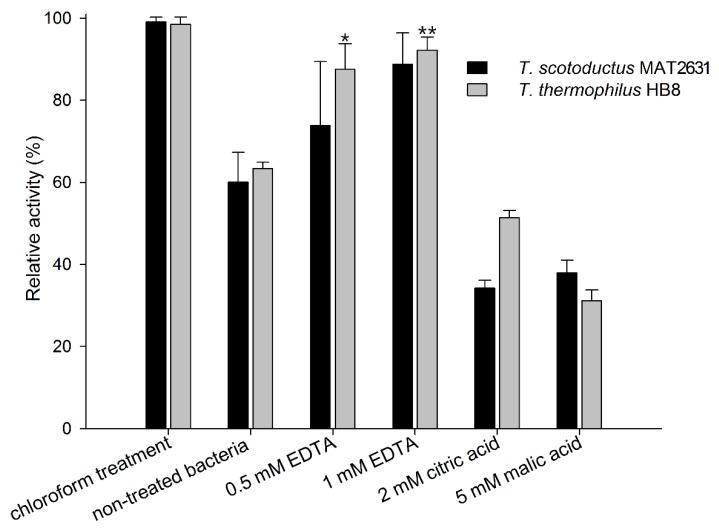
Comparison of the lytic activity of Ts2631 endolysin against chloroform-treated vs. planktonic bacterial substrates in the presence or absence of outer membrane permeabilizers. The activity of Ts2631 endolysin was analysed at 60 °C on planktonic *T. scotoductus* MAT2631 (black bars) and *T. thermophilus* HB8 (grey bars) in the absence (non-treated bacteria) or in the presence of the following outer membrane permeabilizers (OMPs): 0.5 mM EDTA, 1 mM EDTA, 2 mM citric acid, and 5 mM malic acid. The activity of Ts2631 endolysin is indicated as a percentage relative to the activity against chloroform-treated bacterial substrates (chloroform treatment). Negative controls (reaction mixtures with substrate bacteria with or without OMPs) were subtracted from the sample measurement. The experiment was repeated in triplicate; error bars indicate the standard deviation; * *p* = 0.0029; ** *p* = 0.0001; Student’s *t* test.

Purified Ts2631 endolysin was used in turbidity reduction assays to spectrophotometrically measure the decrease in the turbidity of buffer-suspended, viable *Thermus thermophilus* HB8 and *Thermus scotoductus* MAT2631. We used known outer membrane permeabilizers (OMPs): 0.5 mM and 1 mM EDTA, 2 mM citric acid, and 5 mM malic acid [36] to check if those compounds were able to enhance the Ts2631 endolysin activity. Chloroform-treated *T. thermophilus* and *T. scotoductus* cells served as positive controls. The results of the antibacterial activity of Ts2631 endolysin against *T. scotoductus* MAT2631 and *T. thermophilus* HB8 are shown in Figure 1. The experiments were carried out at 60 °C, the temperature optimal for Ts2631 endolysin as well as for its host bacterium [21]. The activity of Ts2631 endolysin against planktonic, viable *Thermus* cells reached an average of 60% in relation to the activity against the chloroform-treated controls (specifically, 60.4 ± 7.3% for *T. scotoductus* and 63.36 ± 1.5% for *T. thermophilus*). Moreover, the addition of EDTA significantly enhanced the activity of Ts2631 endolysin, which reached 87.52 ± 6.3% for 0.5 mM EDTA (*p* = 0.0029) and 92.24 ± 3.2% for 1 mM EDTA (*p* = 0.0001) against *T. thermophilus.* The opposite results were obtained when mild organic acids were tested. The addition of citric and malic acids caused visible cell aggregation, which resulted in a lower than expected Ts2631 endolysin activity against *Thermus* cells in these experimental settings (Figure 1).

### 3.2. Bactericidal Activity of Ts2631 Endolysin against Gram-Negative MDR Bacterial Pathogens

The results showed that Ts2631 endolysin is active against viable thermophilic bacteria at 60 °C, and this activity is further strengthened by the addition of 0.5 mM and 1 mM EDTA. In the next step, we lowered the reaction temperature to 37 °C and checked the antibacterial activity of Ts2631 endolysin against several mesophilic Gram-negative pathogens (Table 1). Interestingly, the addition of Ts2631 endolysin to 10^6^ multidrug-resistant *A. baumannii* KPD 205 caused a 3.71 ± 0.06 log reduction in viable cell counts. With increased bacterial load (10^7^), we still observed significant Ts2631 endolysin activity at the level of 2.18 ± 0.51 logs, which suggests that the endolysin activity is dose dependent on the bacteria. Moreover, Ts2631 endolysin kills *A. baumannii* KPD 205 bacteria at stationary phase of growth (1.66 ± 0.09 logs reduction). The enzyme is not active in the presence of 1× Fetal Bovine Serum. *A. baumannii* KPD 581 was also susceptible to Ts2631 endolysin, with a 2.31 ± 0.04 and 1.07 ± 0.07 log reduction for 10^6^ and 10^7^ bacterial cells, respectively. Lower but still significant Ts2631 endolysin activity was observed in the case of *Pseudomonas aeruginosa* KPD 430 (2.22 ± 0.16 logs) and *P. aeruginosa* KPD 431 (1.36 ± 0.20 logs). On the other hand, bacteria of the Enterobacteriaceae family (*E. coli*, *C. freundii*, *C. braakii*, *K. pneumoniae* and *E. cloacae*) were not sensitive to the Ts2631 endolysin antibacterial activity. The highest reduction within this group was observed for *C. braakii* (0.82 ± 0.06 logs).

Subsequently, we performed Ts2631 endolysin antibacterial tests in the presence of known OM permeabilization agents (EDTA, malic and citric acids). It is noteworthy that the addition of EDTA alone to cell suspensions of 10^7^ cells of each *A. baumannii* and *P. aeruginosa* caused a reduction in the bacterial cell counts by 2.2 and 2.77 logs for *A. baumannii* strains KPD 205 and KPD 581, respectively, and caused the complete eradication of *P. aeruginosa* (Table 2). Overall, the killing effect of Ts2631 endolysin/EDTA has been more pronounced than the killing effect of the combination with malic or citric acid. All enterobacterial strains as well as *Pseudomonas* strains tested were sensitive to Ts2631 endolysin/EDTA treatment. We observed a log reduction of 1.18 ± 0.02 in *K. pneumoniae* and the complete elimination of *C. braakii* (>6 logs). The combination of Ts2631 endolysin with malic acid resulted in a 2.93 ± 0.12 log reduction in *A. baumannii* KPD 205 and a 1.83 ± 0.11 log reduction in *A. baumannii* KPD 581 (these reductions were slightly higher than the reductions after the addition of Ts2631 endolysin alone). Other bacteria tested were not significantly sensitive to Ts2631 endolysin/malic acid treatment. On the other hand, a mixture of Ts2631 endolysin with citric acid resulted in a significant reduction not only in *A. baumannii* KPD 205 (3.30 ± 0.09) and *A. baumannii* KPD 581 (2.16 ± 0.26) but also in *P. aeruginosa* KPD 430 (2.56 ± 0.06), *P. aeruginosa* KPD 431 (1.39 ± 0.05), and *C. braakii* (1.31 ± 0.08 and 1.00 ± 0.05 for 10^6^ and 10^7^ cells, respectively).

In conclusion, the results showed the bactericidal activity of Ts2631 endolysin against alarming MDR pathogens, such as *A. baumannii* and *P. aeruginosa,* and this effect was further enhanced by the OM permeabilization agents. In particular, the addition of 0.5 mM EDTA extended the Ts2631 endolysin spectrum to members of the Enterobacteriaceae family with a reduction in bacterial cell number between 1 and greater than 6 logs.

### 3.3. Mode of Action of Ts2631 Endolysin on ESKAPE Pathogens

**Figure 2:**
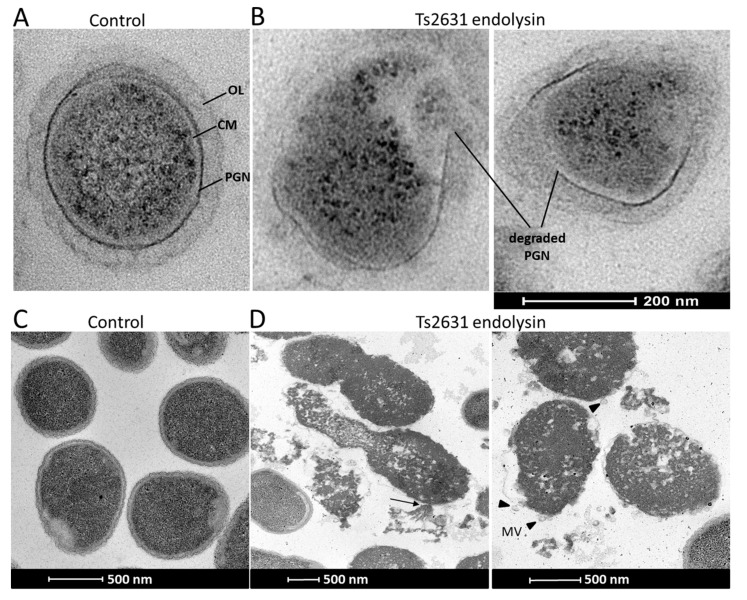
Transmission electron microscopy of *T. thermophilus* HB8 and *A. baumannii* KPD 205 cells treated with Ts2631 endolysin. (**A**) A cross-section of untreated *T. thermophilus* HB8 cells at a scale of 200 nm; (**B**) *T. thermophilus* treated with 7.4 µM of Ts2631 endolysin for 1.5 h. OL, a thick outer layer of amorphous material (30 nm) [37]; PGN, peptidoglycan; CM, cytoplasmic membrane; (**C**) untreated *A. baumannii* cells at a scale of 500 nm; (**D**) *A. baumannii* treated with 7.4 µM of Ts2631 endolysin for 1.5 h. The black arrow indicates the intracellular contents flowing out from the hole of the *A. baumannii* cell envelope. Black arrowheads indicate membrane vesicles (MVs).

Transmission electron microscopy experiments were performed to visualize the antibacterial activity of Ts2631 endolysin (Figure 2). The exposure of 10^8^ viable *T. thermophilus* HB8 cells to Ts2631 endolysin (7.4 µM) for 1.5 h at 60 °C caused the degradation of the peptidoglycan layer of the bacterial cell wall. In cross-sections of rod-shaped *T. thermophilus*, PGN was clearly visible as a dark border between the cytoplasmic membrane and the *T. thermophilus* outer layer (Figure 2A). Upon the addition of Ts2631 endolysin, the PGN layer disintegrated, releasing the cell content (Figure 2B). To unravel the mechanisms behind the bactericidal activity of Ts2631 endolysin at 37 °C, microscopic studies were performed with exponentially growing *A. baumannii* KPD 205 cells. The control showed typical, healthy, coccobacilli-shaped cells with continuous inner and outer membranes and an evenly distributed cytoplasmic content (Figure 2C). In comparison, significant morphological changes were observed in *A. baumannii* KPD 205 cells upon the addition of Ts2631 endolysin (Figure 2D). Our images revealed evidence of cell wall damage with excessive cytoplasmic leakage (see arrow in Figure 2D). Furthermore, a number of bubbles protruded from the cell surface, and irregular stretches of membrane protuberances were visible (black arrowheads in Figure 2D). The cytoplasmic contents of the cells were not evenly distributed and had apparent signs of cell decay.

### 3.4. Intrinsic Membrane Passaging Capability of Ts2631 Endolysin

**Figure 3:**
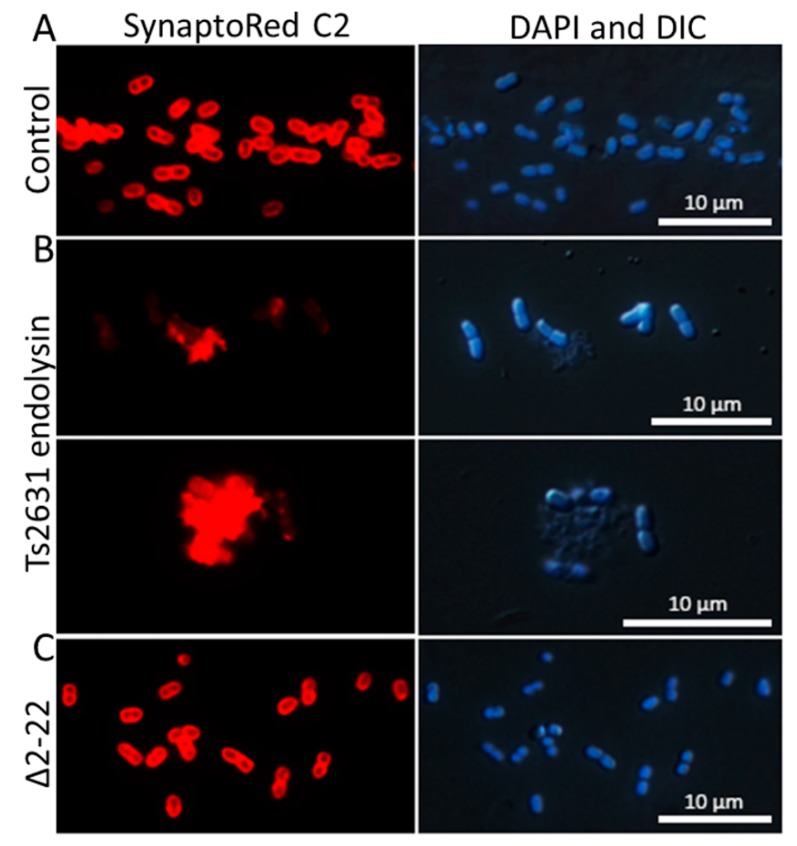
Fluorescence microscopy analysis of *A. baumannii* KPD 205 cells stained with DAPI (nucleoid) and SynaptoRed C2 (membrane). (**A**) *A. baumannii* KPD 205 cells incubated with 10 mM potassium phosphate buffer, pH 8.0. (**B**) Bacteria treated for 1.5 h either with 7.4 µM of Ts2631 endolysin or (**C**) with an equal amount of the Ts2631Δ2–22 variant. After the Ts2631 endolysin treatment, the DAPI and DIC images also showed an unaltered bacterial cell shape, except for the cell debris. These bacteria were not visible by SynaptoRed C2 staining, which indicates extensive bacterial membrane damage. DAPI—4′,6-diamidino-2-phenylindole; DIC—differential interference contrast. Bar indicates 10 μm.

Spectrophotometric observations of the lysis of *T. thermophilus* cells and the results of antibacterial tests (for example, 3.71 ± 0.06 log reduction in *A. baumannii* KPD 205 cells in the presence of endolysin) suggest that Ts2631 has intrinsic antibacterial activity. However, *in silico* analysis using TMHMM v. 2.0 and SignalIP 4.1 predicted neither amphipathic alpha helices nor N-terminal signal-anchor-release domains in the Ts2631 endolysin structure that might be responsible for OM penetration [38,39]; the AMPA web software (http://tcoffee.crg.cat/apps/ampa) was used to assess the antimicrobial protein domains, and the results show that the N-terminal region of Ts2631 endolysin (between amino acids 10 and 31) displays antimicrobial characteristics with only a 2% probability of misclassification [40]. This is in line with our previous observation that the Ts2631Δ2–22 variant, lacking the N-terminal region, does not lyse intact *T. thermophilus* HB8 cells in turbidity reduction assays [22]. The Ts2631Δ2–22 variant was active against chloroform-treated, outer membrane-deprived, bacterial substrates at a level comparable to that of the wild-type protein. To confirm the putative function of the N-terminal region of Ts2631 endolysin in OM penetration, enzyme-treated cells were investigated by fluorescence microscopy with the use of the membrane-binding dye SynaptoRed C2. SynaptoRed C2, which stains bacterial membranes red, enabling us to visualize membrane destruction in our experiments (Figure 3). In our study, we used mesophilic carbapenem-resistant *A. baumannii* KPD 205 cells. Bacteria were treated for 1.5 h with either 7.4 µM of Ts2631 endolysin or an equal amount of the Ts2631Δ2–22 variant and were subsequently subjected to microscopic evaluation. *A. baumannii* KPD 205 cells treated with potassium phosphate buffer, pH 8.0, served as the negative control. 

In the control, coccobacilli-shaped cells were observed with clearly marked, intact bacterial membranes (Figure 3A). In contrast to the control, the cell membranes of Ts2631 endolysin-treated bacteria were not properly stained and were surrounded by many visible cell debris (Figure 3B, SynaptoRed C2). Moreover, images of these cells taken by differential interference contrast microscopy merged with DAPI staining highlighted the proper shape of cells, which was not reflected by SynaptoRed C2 staining (Figure 3B, DAPI and DIC). This indicates that the bacterial envelope damage preceded osmotic lysis, which would be expected due to the mode of action of Ts2631 endolysin. The addition of the Δ2–22 variant to *A. baumannii* was not harmful to the cells and left the bacterial membranes unaltered (Figure 3C). This finding supports the previous observation that the Ts2631Δ2–22 variant, which is fully active in terms of catalytic activity, is not able to lyse bacterial cells that contain an intact OM.

## 4. Discussion

The therapeutic potential of endolysins has been discussed since the first external use of the PlyC lysin of streptococcal bacteriophage C1 to kill group A streptococci in a mouse bacteraemia model [41,42]. Currently, as a result of the joint effort of the scientific community to characterize and to prove the efficacy of endolysins as antibacterial compounds, two phase I clinical trials and two phase I/II clinical trials have been completed [43]. Two phase II clinical studies are now being conducted on patients with *Staphylococcus aureus* bacteraemia with the use of *S. aureus*-specific SAL200 endolysin (N-Rephasin) and CF-301 endolysin, also referred to as PlySs2, derived from the prophage of the *Streptococcus suis* 89/1591 strain [33,42,43,44]. Phage lytic enzymes were classified as the therapeutics with the greatest potential to provide alternatives to antibiotics and are widely considered as safe [5]. One of the encouraging attempts to explore the lytic potential of enzymes derived from extremophilic bacteriophages against Gram-positive bacteria was to study GVE2, the N-acetylmuramoyl-L-alanine amidase of the *Geobacillus* E263 bacteriophage isolated from a deep-sea hydrothermal vent in the east Pacific [45]. The thermostability of the enzyme inspired scientists to use the catalytic domain of GVE2 fused to the cell wall binding domain of the PlyCP26F endolysin from the *C. perfringens*-specific bacteriophage to create an anti-*Clostridium* antimicrobial agent with improved stability [46]. This work is a good example of the successful use of genetic engineering to design chimeric endolysins with improved properties. 

Endolysins used against Gram-positive bacteria outnumber those directed against Gram-negative pathogens [44]. Our study is the first to show the direct bactericidal activity of an endolysin derived from an extremophilic bacteriophage against Gram-negative MDR pathogens. The antibacterial activity of Ts2631 endolysin reported here makes it a good candidate for future domain swap experiments with Gram-negative bacteria.

Fluorescence microscopy experiments (Figure 3) show that the ability of Ts2631 endolysin to pass bacterial OMs is mediated by the naturally occurring N-terminal sequence (residues 1-20) carrying seven positively charged residues (six arginines and one lysine). Although this particular N-terminal portion of the Ts2631 endolysin is not homologous to any other protein sequence present in the UniProt database [22], the targeted enhancement of the bactericidal activity of an endolysin by protein fusion with a cationic peptide has been described in the literature. This can be exemplified by the addition of polycationic nonapeptide (PCNP) to the OBPgp279 endolysin of the *Pseudomonas fluorescens* bacteriophage OBP [47]. Another example is the improved bactericidal effect of the *E. coli* phage endolysin Lysep3 by an increase in the positive charge at its C-terminus [48]. Although rarely, some endolysins display natural intrinsic antibacterial properties. This group contains the aforementioned OBPgp279 endolysin [49], the *Salmonella* phage SPN9CC endolysin [39], the *Bacillus amyloliquefaciens* phage Lys1521 endolysin [50] and endolysins from *Acinetobacter baumannii* phages [51,52,53]. Their specific property relies on the presence of amphipathic α-helices [51], transmembrane domains characteristic of signal-arrest-release endolysins [39] or cationic amino acid motifs [51,52,54], where positively charged amino acid residues interact with negatively charged molecules on the bacterial OM [36,52]. We think that this type of interaction underlies the observed antibacterial activity of Ts2631 endolysin as its molecule contains a positively charged N-terminus that protrudes from the remainder of the enzyme [22].

The antibacterial spectrum of endolysins may be affected by the type and structure of a particular OM. The Ts2631 endolysin has intrinsic antibacterial activities against *Acinetobacter* and *Pseudomonas* but not against the members of the Enterobacteriaceae family. The former have a high number of negatively charged phosphate groups per LPS molecule [36] and, therefore, may be more prone than enterobacteria to destabilizing activity of the positively charged N-terminal extension of Ts2631 endolysin. On the other hand, highly negatively charged OMs can interact with a greater number of divalent cations present in the surrounding milieu. In the case of *Pseudomonas*, this contributes to greater rigidity and tightness of the OM [55] but sensitizes the outer membrane to EDTA, which is a common divalent cation chelator and chelates cations, such as Mg^2+^ and Ca^2+^. This explains the sensitivity of *Pseudomonas* to 1 mM EDTA (>6 log reduction in viable cell number) in contrast to the sensitivity of Enterobacteriaceae, which are resistant to EDTA concentrations up to 5 mM [36].

As a consequence, endolysin-treated *Pseudomonas* cells become round and burst due to osmotic imbalance, releasing the cell contents and membrane fragments, which rapidly form membrane vesicles (MVs) [56,57]. Our observations of *A. baumannii* cytolysis after the addition of Ts2631 endolysin indicate a similar mechanism. The formation of membrane vesicles is clearly visible, but changes in the bacterial shape are not obvious, perhaps because of the coccobacilli shape rather than the rod shape of *Acinetobacter* cells. The cytolysis of *A. baumannii* has been observed under electron microscopy and is clearly different from that described for the Gram-positive *Bacillus anthracis* cells, where the cytoplasmic membrane is extruded through the holes in the peptidoglycan layer and forms the cytoplasmic blebs; however, the cell wall does not entirely disintegrate [58].

The discovery of new antibiotics effective against Gram-negative bacteria is a major challenge, and the rate of success is almost 1000-fold lower for antibiotics against *P. aeruginosa* than for those against Gram-positive bacteria [59]. Hopefully, the feasibility of the external use of Ts2631 endolysin to kill Gram-negative bacterial pathogens presented in our report will draw attention to the previously omitted extremophilic lytic enzymes as potential antimicrobials.

## Figures and Tables

**Table 1:** Bactericidal activity of Ts2631 against several Gram-negative bacterial pathogens. The significant log reduction units observed (≥1 log kill) are marked in bold.

Bacterial Species	Ts2631	Origin/Characteristics ^#^
*A. baumannii* CRAB KPD 205	**3.71 ± 0.06** 2.175 ± 0.51 * 1.66 ± 0.09 **	Carbapenem-resistant clinical strain (PIP, TZP, CAZ, FEP, IMP, MEM, CIP, LVX, and SXT)
*A. baumannii* MDR KPD 581	**2.31 ± 0.04** 1.07 ± 0.07 *	Multidrug-resistant clinical strain (AMP, AMC, TZP, CEP, CXM, FOX, CTX, CAZ, FEP, ETP, MEM, CIP, SXT, and TOB)
*P. aeruginosa* KPD 430	**2.22 ± 0.16**	Clinical strain (PIP, TZP, CAZ, FEP, CIP, LVX, TCC, and TOB)
*P. aeruginosa* CRPA KPD 431	**1.36 ± 0.20**	Clinical strain (GM, PIP, TZP, CAZ, FEP, TCC, and MEM)
*E. coli* KPD 217	0.35 ± 0.08	Clinical strain (AMP, AMC, TZP, CEP, CXM, FOX, CTX, CAZ, FEP, CSL, and SXT)
*C. freundii* KPD 219	0.00	Clinical strain (AMP, AMC, TZP, CEP, CXM, FOX, CTX, CAZ, FEP, CSL, ETP, and SXT)
*C. braakii* KPD 218	0.82 ± 0.06 0.63 ± 0.06 *	Clinical strain (AMP, AMC, TZP, CEP, CXM, FOX, CTX, CAZ, FEP, CSL, ETP, and SXT)
*K. pneumoniae* KPD 298	0.11 ± 0.03	Clinical strain (AMP, AMC, TZP, CEP, CXM, FOX, CTX, CAZ, FEP, ETP, IMP, MEM, AKN, CIP, SXT, and TOB)
*E. cloacae* KPD 297	0.00	Clinical strain (AMP, AMC, TZP, CEP, CXM, FOX, CTX, CAZ, FEP, CSL, ETP, CIP, and SXT)

**^#^** Patterns of antibiotic resistance provided by the Department of Clinical Microbiology at University Clinical Centre, Gdansk, Poland, based on susceptibility tests performed according to the EUCAST for antibiotic resistance; PIP, Piperacillin; TZP, Piperacillin/tazobactam; CAZ, Ceftazidime; FEP, Cefepime; IMP, Imipenem; MEM, Meropenem; CIP, Ciprofloxacin; LVX, Levofloxacin; SXT, Trimethoprim/sulfamethoxazole; AMP, Ampicillin; AMC, Amoxicillin/clavulanic acid; AKN, amikacin; CEP, Cephalothin; CXM, Cefuroxime sodium; FOX, Cefoxitin; CTX, Cefotaxime; CSL, Cefoperazone/sulbactam; ETP, Ertapenem; TCC, Ticarcillin/clavulanic acid; TOB, Tobramycin; GM, Gentamicin. The asterisk indicates a log reduction of 10^7^ bacterial cells used in the antibacterial assay; two asterisks indicate log reduction of 10^6^ bacterial cells at stationary phase of growth at OD600 = 1.2.

**Table 2:** Combined bactericidal activity of the Ts2631/outer membrane permeabilizers (EDTA, citric and malic acid) against several Gram-negative multidrug-resistant (MDR) bacterial pathogens. The significant log reduction units observed (≥1 log kill) are marked in bold.

Bacterial Species	K/PO_4_/EDTA	Ts2631/EDTA	K/PO_4_/Malic	Ts2631/Malic	K/PO_4_/Citric	Ts2631/Citric
*A. baumannii* CRAB* KPD 205	**2.20 ± 0.05**	**4.24 ± 0.40**	0.00	**2.93 ± 0.12**	0.00	**3.30 ± 0.09**
*A. baumannii* MDR* KPD 581	**2.77 ± 0.05**	**3.93 ± 0.05**	0.00	**1.83 ± 0.11**	0.00	**2.16 ± 0.26**
*P. aeruginosa* KPD 430	**>6.00**		0.00	0.81 ± 0.20	0.00	**2.56 ± 0.06**
*P. aeruginosa* CRPA KPD 431	**>6.00**		0.00	0.58 ± 0.14	0.00	**1.39 ± 0.05**
*E. coli* KPD 217	0.68 ± 0.07	**3.08 ± 0.11**	0.00	0.65 ± 0.16	0.00	0.98 ± 0.08
*C. freundii* KPD 219	0.78 ± 0.06	**3.45 ± 0.37**	0.00	0.00	0.00	0.05 ± 0.02
*C. braakii* KPD 218	0.94 ± 0.07	**>6.00**	0.25 ± 0.12	0.94 ± 0.07	0.33 ± 0.14	**1.31 ± 0.08**
*C. braakii* KPD 218 *	0.84 ± 0.08	**5.47 ± 0.08**	0.00	0.77 ± 0.18	0.00	**1.00 ± 0.05**
*K. pneumoniae* KPD 298	0.24 ± 0.09	**1.18 ± 0.02**	0.00	0.00	0.00	0.00
*E. cloacae* KPD 297	0.40 ± 0.13	**3.85 ± 0.04**	0.00	0.00	0.00	0.14 ± 0.02

The asterisk indicates a log reduction of 10^7^ bacterial cells used in the antibacterial assay.
